# DNA-binding residues and binding mode prediction with binding-mechanism concerned models

**DOI:** 10.1186/1471-2164-10-S3-S23

**Published:** 2009-12-03

**Authors:** Yu-Feng Huang, Chun-Chin Huang, Yu-Cheng Liu, Yen-Jen Oyang, Chien-Kang Huang

**Affiliations:** 1 Department of Computer Science and Information Engineering, National Taiwan University, Taipei, 106, Taiwan, Republic of China; 2 Department of Engineering Science and Ocean Engineering, National Taiwan University, Taipei, 106, Taiwan, Republic of China; 3 Institute of Biomedical Engineering, National Taiwan University, Taipei, 106, Taiwan, Republic of China; 4 Graduate Institute of Biomedical Electronics and Bioinformatics, National Taiwan University, Taipei, 106, Taiwan, Republic of China; 5 Center for Systems Biology and Bioinformatics, National Taiwan University, Taipei, 106, Taiwan, Republic of China

## Abstract

**Background:**

Protein-DNA interactions are essential for fundamental biological activities including DNA transcription, replication, packaging, repair and rearrangement. Proteins interacting with DNA can be classified into two categories of binding mechanisms - sequence-specific and non-specific binding. Protein-DNA specific binding provides a mechanism to recognize correct nucleotide base pairs for sequence-specific identification. Protein-DNA non-specific binding shows sequence independent interaction for accelerated targeting by interacting with DNA backbone. Both sequence-specific and non-specific binding residues contribute to their roles for interaction.

**Results:**

The proposed framework has two stage predictors: DNA-binding residues prediction and binding mode prediction. In the first stage - DNA-binding residues prediction, the predictor for DNA specific binding residues achieves 96.45% accuracy with 50.14% sensitivity, 99.31% specificity, 81.70% precision, and 62.15% F-measure. The predictor for DNA non-specific binding residues achieves 89.14% accuracy with 53.06% sensitivity, 95.25% specificity, 65.47% precision, and 58.62% F-measure. While combining prediction results of sequence-specific and non-specific binding residues with OR operation, the predictor achieves 89.26% accuracy with 56.86% sensitivity, 95.63% specificity, 71.92% precision, and 63.51% F-measure. In the second stage, protein-DNA binding mode prediction achieves 75.83% accuracy while using support vector machine with multi-class prediction.

**Conclusion:**

This article presents the design of a sequence based predictor aiming to identify sequence-specific and non-specific binding residues in a transcription factor with DNA binding-mechanism concerned. The protein-DNA binding mode prediction was introduced to help improve DNA-binding residues prediction. In addition, the results of this study will help with the design of binding-mechanism concerned predictors for other families of proteins interacting with DNA.

## Background

Protein-DNA interactions play important roles for the regulation of key biological functions like DNA transcription, replication, packaging and recombination. With the increasing number of high quality structure of complexes in Protein Data Bank (PDB) [1] and Nucleic Acid Database (NDB) [2], the collection of atomic interaction information for their structural and functional integrity is sufficiently complete for analysis and prediction of protein-nucleic acid interactions. Atomic level analyses have been investigated to understand how amino acids interact with nucleotide bases or sugar-phosphate backbones through hydrogen bonds, van der Waals contacts, or water-mediated hydrogen bonds [3], depending on the amino acid propensities [4,5]. In recent years, the prediction of residues in a protein chain that interact with DNA has been a research topic that attracts a high level of interest. Some of the studies were purely based on analysis of the protein polypeptide sequence [6-11], while the others took the structural information into account [12-17]. Particularly, the issue for sequence-specific binding residue prediction has been also mentioned recently [18]. Transcription factors (TFs) are proteins that regulate gene expression, which serve as integration centers of the different signal-transduction pathways affecting a given gene [19]. TFs regulate cell development, differentiation, and cell growth by binding to a specific DNA site and regulating gene expression [20-22]. As it has been reported in a recent article that the tertiary structures of a large number of TFs are mostly disordered [23], sequence based analysis aimed at identifying the residues in a highly-disordered TF that play key roles in interaction with the DNA is essential for obtaining a comprehensive picture of how TFs function.

As studied in previous research, proteins that interact with DNA will change their conformations from their free states, changing non-specific complexes to specific complexes [24]. During the course of DNA-recognition, residues play different roles to either recognize nucleotide bases or stabilize the protein-DNA conformation. In this work, we try to identify whether the residue performs sequence-specific or non-specific binding. There are two types of binding mechanisms involved in amino acid - nucleotide interactions, namely sequence-specific and non-specific site binding [25-29]. Sequence-specific binding occurs between protein side-chains and nucleotide bases, while non-specific binding occurs between protein side-chains and the DNA sugar/phosphate backbone [28]. In general, sequence-specific binding is also named as specific binding. Specific binding corresponds to sequence-specific recognition of a gene and therefore is essential for the correct regulation of genes. Non-specific binding shows relatively little base-sequence preference and binds preferentially to either single or double-stranded DNA. The role for non-specific binding residues is to stabilize the interactions between protein and nucleotide backbone to help specific binding residues in recognizing base pairs correctly. As reported in the review article by Luscombe *et al. *[30], protein-DNA interactions can be grouped into eight different structural/functional groups based on the structures of the DNA-binding region in the proteins, which is also referred to as the binding mode of the protein [30-32]. There are eight such binding modes including (I) Helix-Turn-Helix, HTH (including "winged" HTH), (II) zinc-coordinating, (III) zipper-type, (IV) other *α-*helix, (V) *β-*sheet, (VI) *β*-hairpin/ribbon, (VII) other, (VIII) enzymes. Related research has investigated the classification of protein-DNA complexes and structural domains [33-35]. Proteins in the same class have similar binding site conformations despite having different DNA targets. The importance of introducing the DNA-binding mode information is to find the binding pattern that a protein uses to interact with the target DNA [36,37], which could help to identify the location of sequence-specific and non-specific binding residues.

**Figure 1:**
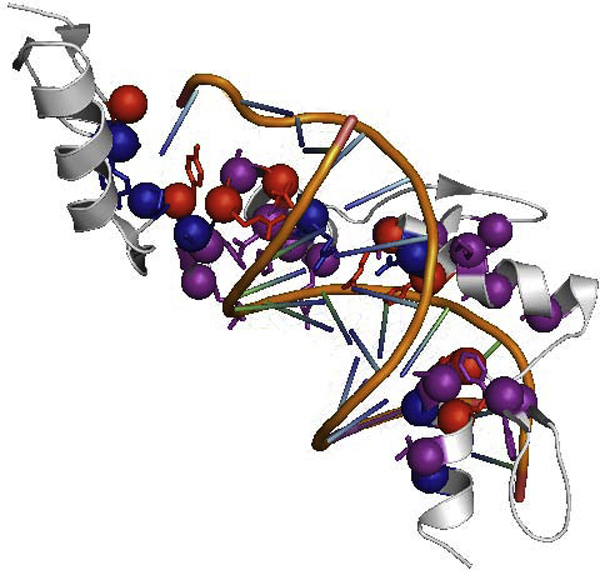
**Sequence-specific and non-specific binding residues of PDB **2PRT:A. Residues colored by red are sequence-specific binding residues. Residues colored by blue are non-specific binding residues. Residues colored by purple are both sequence-specific and non-specific binding residues.

This article presents the design of a sequence based predictor for identifying the residues in a TF that are involved in both sequence-specific binding and non-specific binding with the DNA and the binding mode. We use support vector machine (SVM) as the classifier to predict binding residues as sequence-specific or non-specific according to binding specificity. Originally, the definition of sequence-specific binding and the non-specific binding residues is based on the identification of hydrogen bonds and van der Waals attractions between protein side-chains and DNAs. In this work, we use a computational approximation of distance cut-off to define binding classification instead. A residue is regarded as involved in sequence-specific binding with the DNA if one or more heavy atoms on its side-chain are within 4.5 Å from any of the nucleic bases, while a residue is regarded as involved in non-specific binding with the DNA if one or more heavy atoms on its side-chain are within 4.5 Å from the sugar/phosphate backbone of the DNA. The threshold of distance cut-off is based on hydrogen bonding and van der Waals attractions: (1) a hydrogen bond was defined as having a maximum donor acceptor distance of 3.35 Å and maximum hydrogen-acceptor distance of 2.7 Å. (2) atoms were considered to form van der Waals contacts if the distance between them was ≤ 3.9 Å and the contact had not been defined as a hydrogen bond [5]. Residues in a protein interacting with DNA play their roles on specific binding, or non-specific binding, or both. The reason to predict both sequence-specific and non-specific binding residues is that the main determinants of specificity are the unfavorable contributions of "wrong" base pairs and specific binding will also require a large non-specific contribution to the binding free energy to achieve sufficient binding affinity [38]. Furthermore, the information of the predicted sequence-binding and non-specific binding residues can be used protein-DNA binding mode prediction. As shown in Figure 1, this is an example of PDB ID 2PRT:A to show sequence-specific and non-specific binding residues in the tertiary structure. Residues colored by red for sequence-specific binding residues, blue for non-specific binding residues, and purple for both sequence-specific and non-specific binding residues.

## Results and discussion

In this section, we will report the experiments conducted to evaluate the performance of our proposed approach. In the experiments of the first stage, we repeated the same testing procedure 20 times with randomly and independently generated testing data sets. The independent testing data set used in each run was derived from 30 TF chains randomly selected from the 253 TF-DNA complexes that we have collected (see Materials and Methods for details). In order to eliminate possible bias present in our collection of TF complexes, we took steps to guarantee that no two TF chains used to generate the testing data set in the same run are homologous with a sequence identity higher than 20%. Furthermore, aiming to obtain experimental results that accurately reflect the actual performance observed by the users of our proposed approach, we guaranteed that the training data generated with a TF chain that is homologous to the protein chain under testing by having a sequence identity higher than 20% are removed. For this study, LIBSVM http://www.csie.ntu.edu.tw/~cjlin/libsvm was used for data training and classification [39]. Table 1 shows the overall performance of the SVM predictor for predicting sequence-specific and non-specific binding residues in the first stage. The results have been obtained using the training parameters, C = 2^2^, γ = 2^-5^, class weight for binding residue is 1.5, and class weight for non-binding residue is 1, which give better results than other values for prediction of sequence-specific binding residues. The predictor for DNA specific binding residues achieves 96.45% accuracy with 50.14% sensitivity, 99.31% specificity, 81.70% precision, and 62.15% F-measure. The results have been obtained using the training parameters, C = 2^0^, γ = 2^-5^, class weight for binding residue is 2, and class weight for non-binding residue is 1, which give better results than other values for prediction of non-specific binding residues. The predictor for DNA non-specific binding residues achieves 89.14% accuracy with 53.06% sensitivity, 95.25% specificity, 65.47% precision, and 58.62% F-measure. While combining prediction results of sequence-specific and non-specific binding residues with OR operation, the predictor achieves 89.26% accuracy with 56.86% sensitivity, 95.63% specificity, 71.92% precision, and 63.51% F-measure. Table 2 shows the breakdown of overall performance of the binding residues prediction in terms of secondary structure elements. The number of sequence-specific (or non-specific) binding residues in β-sheet secondary structure elements is far fewer than the number of sequence-specific (or non-specific) binding residues in either α-helix or coil elements. As a result, our proposed framework cannot learn sufficient clues in order to identify sequence-specific (or non-specific) binding residues in β-sheet elements.

**Table 1:** Overall performance of proposed approach

Binding type	# of residues	TP	FP	TN	FN	Sensitivity	Specificity	Precision	Accuracy
**Sequence-specific binding**	60466	1764	395	56553	1754	50.14%	99.31%	81.70%	96.45%
**Non-specific binding**	60466	4652	2454	49245	4115	53.06%	95.25%	65.47%	89.14%
**Specific+Non-specific**	60466	5651	2206	48321	4288	56.86%	95.63%	71.92%	89.26%

**Table 2:** Performance of the binding site prediction in terms of secondary structure elements

Binding type	Secondary structure elements	# of residues	TP	FP	TN	FP	Sensitivity	Specificity	Precision	Accuracy
**Specific**	**Helix**	32670	1322	279	30160	909	59.26%	99.08%	82.57%	96.36%
	**Sheet**	5259	22	0	5077	160	12.09%	100.00%	100.00%	96.96%
	**Coil**	22537	420	116	21316	685	38.01%	99.46%	78.36%	96.45%

**Non-specific**	**Helix**	32670	2197	1005	27458	2010	52.22%	96.47%	66.61%	90.77%
	**Sheet**	5259	257	185	4524	293	46.73%	96.07%	58.15%	90.91%
	**Coil**	22537	2198	1264	17263	1812	54.81%	93.18%	63.49%	86.35%

**Specific + Non-specific**	**Helix**	32670	2988	858	26783	2041	59.42%	96.90%	77.69%	91.13%
	**Sheet**	5259	261	181	4472	345	43.07%	96.11%	59.05%	90.00%
	**Coil**	22537	2402	1167	17066	1902	55/81%	93.60%	67.305	86.38%

**Figure 2:**
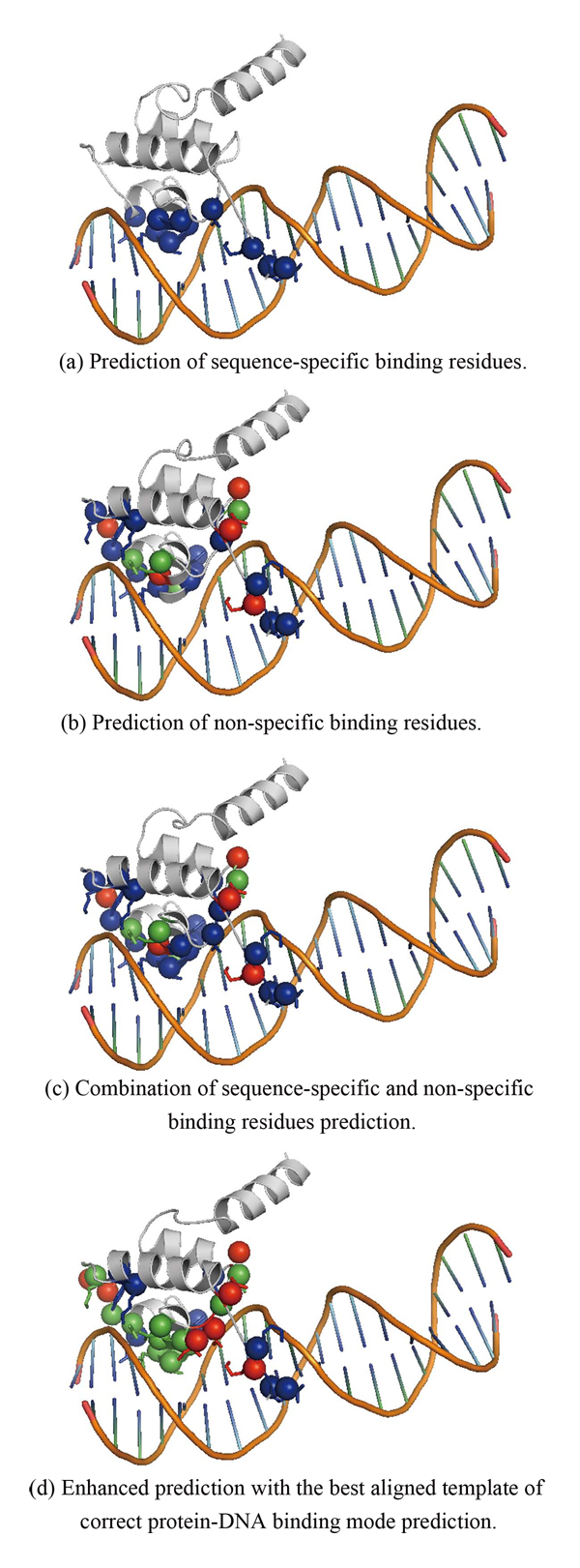
**A difficult case (PDB ID **1LMB:4**) of binding residue prediction, which can be enhanced with the best aligned template of correct predicted protein-DNA binding mode**. Residues colored by red means false positive. Residues colored by blue means false negative. Residues colored by green means true positive. (a) Prediction of sequence-specific binding residues. (b) Prediction of non-specific binding residues. (c) Combination of sequence-specific and non-specific binding residues prediction. (d) Enhanced prediction with the best aligned template of correct protein-DNA binding mode prediction.

In the experiments of the second stage, the protein-DNA binding mode prediction achieves 75.83% overall accuracy while applying LIBSVM with multi-class prediction using one-against-one approach. As shown in Table 3, the predictor can deliver precision of 100% and sensitivity of 80.22% for zipper-type binding mode, precision of 70.45% and sensitivity of 73.46% for helix-turn-helix binding mode, precision of 68.07% and sensitivity of 88.98% for zinc-coordinating binding mode, and precision of 34.21% and sensitivity of 52.00% for β-hairpin/ribbon binding mode. The predictor did not perform well for TFs with a binding mode of β-hairpin/ribbon. The reason is that the prediction power of sequence-specific binding and non-specific binding residue on β-sheet structure is worse than that of α-helix and coil. We select PDB 1LMB:4 as an example to show how the predicted binding mode information can be used to enhance the binding residues prediction. Figure 2 displays the prediction result of PDB ID 1LMB:4, which is a difficult case in our binding residues prediction experiment. The protein, 1LMB:4, belongs to the HTH_3 domain which is classified in the group of helix-turn-helix, which has 10 sequence-specific binding residues and 18 non-specific binding residues. However, the predictor found no sequence-specific binding residues with 10 false negatives and found 4 non-specific binding residues with 14 false negatives and 5 false positives. The binding mode predictor can correctly classify the 1LMB:4 into helix-turn-helix group. According to the best alignments of secondary structure elements, a protein is selected from the helix-turn-helix group. In Figure 2, residues are colored by red for false positive, blue for false negative and green for true positive. Figure 2(a), 2(b), 2(c) show the prediction of sequence-specific binding residues, the prediction of non-specific binding residues, and the combined result, respectively. Figure 2(d) shows the enhanced prediction with the best aligned template of correct protein-DNA binding mode prediction. It is obviously that correct binding mode prediction can greatly help the binding residues prediction, especially in difficult case. However, this idea needs more investment to derive a systematic approach.

**Figure 3:**
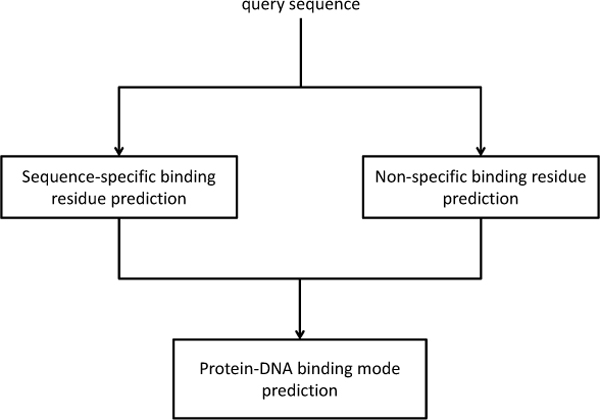
**Overall framework for DNA-binding residues prediction**.

**Table 3:** Overall performance of protein-DNA binding mode prediction

Protein-DNA binding mode	# of protein chains	Sensitivity	Precision
**zipper-type**	146	100.00%	80.22%
**helix-turn-helix (HTH)**	220	70.45%	73.46%
**zinc-coordinating**	166	68.07%	88.98%
**β-hairpin/ribbon**	38	34.21%	52.00%
**others**	30	93.33%	50.91%

In the following section, we will discuss how the proposed approach performs in comparison with the related studies reported in recent years. One must note that our proposed approach is the only predictor listed in Table 4 that identifies the residues involved in both sequence-specific and non-specific binding with DNA, while all the other predictors do not distinguish between sequence-specific binding and non-specific binding. Since the results listed in Table 4 include the main results extracted from recent studies along with the overall results with our proposed approach, it should be regarded as a survey of the latest advances in the field. It must also be noted that most related studies have adopted slightly different definitions of DNA-binding residues. In the article by Ahmad and Sarai [10] and in the article by Wang and Brown [40], a residue is regarded as involved in interaction with the DNA if one of its heavy atom is within 3.5 Å from a heavy atom of the DNA. In the article by Hwang *et al.*, a larger threshold of 4.5 Å is used instead of 3.5 Å. In the article by Yan *et al. *[8], a residue is regarded as involved in interaction with the DNA if its solvent accessible surface area (ASA) in the protein-DNA complex is less than its ASA in the unbound protein by more than 1 Å^2^.

**Table 4:** Performance delivered by alternative predictors of DNA-binding residues, where the F-measure is the harmonic mean of precision and sensitivity

Predictor	Sensitivity	Specificity	Accuracy	Precision	F-measure
**Sequence-specific binding**	0.501	0.993	0.965	0.817	0.622
**Non-specific binding**	0.530	0.953	0.891	0.655	0.586
**Specific+Non-specific**	0.569	0.956	0.893	0.719	0.635
**Ahmad and Sarai **[10]	0.682	0.660	0.664	0.308*	0.425*
**Yan et al. **[8]	0.410	0.871	0.780	0.439*	0.424*
**BindN (Wang and Brown) **[40]	0.652	0.728	0.722	0.186*	0.289*
**DP-Bind (Hwang et al.) **[7]	0.791	0.786	0.800	-*	-*

*The numbers with an asterisk are those that have been derived from the numbers reported in the related studies.

The numbers listed in Table 4 with an asterisk have been derived from the numbers reported in the related studies. Since all the four related studies addressed in Table 4 reported three out of the four performance metrics listed in the table, we can obtain 3 equations about the following 4 variables for each of the related study:

In addition, we have . Therefore, for each related study, we can derive the actual value of the fourth performance metric based on the values of the other three performance metrics that were provided. The only exception is precision for the predictor proposed by Hwang *et al. *[7]. By definition, the accuracy cannot be higher than the sensitivity and the specificity simultaneously, which is the case with the numbers reported by Hwang *et al. *Therefore, there is no way to derive the exact value of precision for their predictor.

According to the observation of the predicted results, the predictor of non-specific binding residues tries to locate positive charged patches. However, not all positive charged patches in a protein will come into contact with single- or double-strand DNA. It might be the reason of the performance gap between sequence-specific and non-specific binding residue prediction. While combining prediction results of sequence-specific and non-specific binding residues, sensitivity is higher than other predictors. The reason is that non-specific binding residues help a protein to slide along the target DNA, and specific binding residues will recognize base pairs while sliding along the target DNA. The role the non-specific binding residues play is to help specific binding residues recognize base pairs precisely. Therefore, the prediction of non-specific binding residues can increase the predictor's capability for predicting DNA-binding residues.

## Conclusion

This article presents the design of a sequence based predictor that aims to identify the sequence-specific and non-specific DNA-binding residues in a TF. As a recent study has revealed that the tertiary structures of a large number of transcription factors are mostly disordered, a sequence based predictor is essential for analyzing how a TF interacts with DNA. Furthermore, it is highly desirable to have a predictor capable of identifying the residues involved in sequence-specific binding with DNA, since sequence-specific binding corresponds to sequence-specific recognition of a gene and is therefore essential for correct gene regulation. However, non-specific binding residues can help specific binding residues to increase binding specificity as well.

In the experiments reported in this article, our proposed approach has been able to deliver precision 81.70% and 65.47% in sequence-specific and non-specific binding residue prediction respectively. Precision of 81.70% implies that about 4 out of 5 predicted binding residues are really involved in sequence-specific binding with the DNA. Precision of 65.47% implies that about 7 out of 10 predicted binding residues are really involved in non-specific binding with the DNA. While combining prediction results, the performance for DNA-binding residue prediction can deliver sensitivity 56.85%. Sensitivity of 56.85% implies that our proposed approach can catch about 6 out of 10 residues involved in DNA binding with the DNA. In the DNA-binding segment of the protein, regions where non-specific binding residues are located will cover the regions where specific binding residues are located. Therefore, improvement can be achieved for DNA-binding residues prediction while combining prediction results of specific and non-specific binding residues. The protein-DNA binding mode prediction is also proposed in this framework, and we select 1LMB:4 as an example to reveal how can be helpful for improving DNA-binding residue prediction.

It is anticipated that the prediction accuracy delivered by our proposed approach will continue to improve as the number of TF-DNA complexes deposited in the PDB continues to grow which will increase the number of training samples for use in our learning algorithm. Nevertheless, the primary interest of computational biologists is to develop more advanced prediction mechanisms. In this respect, we believe that as the number of TF-DNA complexes deposited in the PDB increases, we can obtain more insights about the key physiochemical properties that play essential roles in TF-DNA interactions to be used to develop more advanced prediction mechanisms. In addition, we will exploit the experiences learned in this study in order to design binding-mechanism concerned predictors for other families of proteins interacting with DNA. We believe that different families of proteins may have very different characteristics. Therefore, a specifically-designed predictor should be created for each specific type of protein to be able to deliver superior performance in comparison with a general-purpose predictor.

## Materials and methods

### Datasets

Our analysis was based on the dataset of DNA-binding residue prediction collected by Ofran and Rost [6]. In this collection, there are 691 protein-DNA complexes. Because we focus on transcription factors, we have created a data set containing 253 TF-DNA complexes among which 227 complexes were extracted from the 691 protein-DNA complexes, and the remaining 26 TF-DNA complexes are those that were deposited into PDB between September 2007 and November 2008. All protein structures are determined by X-ray crystallization at a resolution of 3.5 Å or better. Using the Gene Ontology (GO) terms [41], we use proteins where the molecular function is transcription factor activity, biological process is transcription, and cellular component is nucleus to select transcription factors. All 253 TF-DNA complexes are listed in Table 5.

**Table 5:** Dataset of 253 TF-DNA complexes for DNA-binding residues prediction

253 TF-DNA Complexes									
1A02:F	1A02:J	1A0A:A	1A0A:B	1A6Y:A	1A6Y:B	1AKH:A	1AKH:B	1AM9:A	1AM9:B
1AM9:C	1AM9:D	1AN2:A	1AN4:A	1AN4:B	1APL:C	1APL:D	1AU7:A	1AU7:B	1B01:A
1B01:B	1B72:B	1B8I:B	1BDT:A	1BDT:B	1BDT:C	1BDT:D	1BDV:A	1BDV:B	1BDV:C
1BDV:D	1BY4:A	1BY4:B	1BY4:C	1BY4:D	1C0W:A	1C0W:B	1C0W:C	1C0W:D	1CF7:A
1CF7:B	1CGP:A	1CGP:B	1CMA:A	1CMA:B	1CQT:A	1D5Y:A	1D5Y:B	1D5Y:C	1D5Y:D
1D66:A	1D66:B	1DDN:A	1DDN:B	1DDN:C	1DDN:D	1DSZ:A	1DSZ:B	1DU0:A	1DU0:B
1EA4:A	1EA4:B	1EA4:D	1EA4:E	1EA4:F	1EA4:G	1EA4:H	1EA4:J	1EA4:K	1EA4:L
1F2I:G	1F2I:H	1F2I:I	1F2I:J	1F2I:K	1F2I:L	1F5T:A	1F5T:B	1F5T:C	1F5T:D
1FJL:A	1FJL:B	1FJL:C	1FOS:E	1FOS:F	1FOS:G	1FOS:H	1G2D:C	1G2D:F	1G2F:C
1G2F:F	1GDT:A	1GDT:B	1H88:A	1H88:B	1H89:A	1H89:B	1H8A:A	1H8A:B	1H9T:A
1H9T:B	1HCQ:A	1HCQ:B	1HDD:C	1HDD:D	1HF0:A	1HF0:B	1HJB:A	1HJB:B	1HJB:D
1HJB:E	1HLO:A	1HLO:B	1HW2:A	1HW2:B	1HWT:C	1HWT:D	1HWT:G	1HWT:H	1IO4:A
1IO4:B	1JGG:A	1JGG:B	1JNM:A	1JNM:B	1JT0:A	1JT0:B	1JT0:C	1JT0:D	1JWL:A
1JWL:B	1K61:A	1K61:B	1K61:C	1K61:D	1KB2:A	1KB2:B	1KB4:A	1KB4:B	1KB6:A
1KB6:B	1KU7:A	1L3L:A	1L3L:B	1L3L:C	1L3L:D	1LAT:A	1LAT:B	1LB2:A	1LE8:A
1LE8:B	1LLI:A	1LLI:B	1LLM:C	1LMB:3	1LMB:4	1MDY:A	1MDY:C	1MDY:D	1MEY:C
1MEY:F	1MJM:A	1MJM:B	1MJP:A	1MJP:B	1MNM:C	1MNM:D	1NKP:A	1NKP:B	1NKP:D
1NKP:E	1NLW:A	1NLW:B	1NLW:D	1NLW:E	1P47:A	1P47:B	1PAR:A	1PAR:B	1PAR:C
1PAR:D	1PER:L	1PER:R	1PUF:A	1PUF:B	1PYI:A	1PYI:B	1QP9:A	1QP9:B	1QP9:C
1QP9:D	1R0N:A	1RPE:L	1RPE:R	1TF6:A	1TF6:D	1TRO:A	1TRO:C	1TRO:E	1TRO:G
1TRR:A	1TRR:B	1TRR:D	1TRR:E	1TRR:G	1TRR:H	1TRR:J	1TRR:K	1YRN:A	1YRN:B
1YSA:C	1YSA:D	1ZME:C	1ZME:D	2DRP:A	2DRP:D	2HAP:C	2HAP:D	2HDD:A	2HDD:B
2NLL:A	2NLL:B	2OR1:L	2OR1:R	2PRT:A	2QL2:A	2QL2:B	2QL2:C	2QL2:D	2R5Y:A
2R5Y:B	3BPY:A	3CBB:A	3CBB:B	3CO6:C	3COQ:A	3COQ:B	3D0A:A	3D0A:B	3D0A:C
3D0A:D	3DFX:A	3DFX:B	3DZY:A	3DZY:D	3E00:A	3E00:D	3EXJ:A	3EXJ:B	3EXL:A
3HDD:A	3HDD:B	9ANT:A							

### Defining the DNA-binding residue

Previous research used various distance cut-offs from 3.5 Å to 6 Å to define DNA-binding residues between proteins and DNA [6-10,14,40,42]. Most, if not all, of the cut-off distance is measured between the atoms of amino acid and the atoms of nucleotide bases or sugar-phosphate backbones. Most DNA-binding residue prediction tools used 3.5 Å or 4.5 Å as the distance cut-off in general. Considering electrostatic interaction, hydrogen bonding, water-mediated hydrogen bonding, and van der Waals contacts, we use 4.5 Å distance cut-off to label DNA-binding residues. A residue is regarded as involved in sequence-specific binding with DNA if one or more heavy atoms on its side-chain are within 4.5 Å from the nucleic bases of the DNA. A residue is regarded as involved in non-specific binding with the DNA, if one or more heavy atoms on its side-chain are within 4.5 Å from the sugar/phosphate backbone of the DNA. In all 253 TF-DNA complexes, there are 1526 binding residues and 23371 non-binding resides for sequence-specific binding residue prediction. The ratio of positive to negative samples is 1:15 in sequence-specific binding. For non-specific binding residue prediction, there are 3831 binding residues and 21066 non-binding residues. The ratio of positive to negative samples is 1:5 in non-specific binding. The number of non-specific binding residues is twice as many as the number of sequence-specific binding residues. Without distinguishing between sequence-specific and non-specific binding residues, there are 4360 binding residues and 20537 non-binding residues. All missing residues which do not have coordinate information in the PDB data file, will be excluded from the training and testing datasets.

### Framework of DNA-binding residues and binding mode prediction using support vector machine

We proposed the two stage framework to predict the DNA-binding residues in a protein and the corresponding binding mode for a query protein respectively. Figure 3 shows the overall framework for binding residue prediction and a binding mode prediction. The first stage predicts the DNA binding residues and the second stage predicts the protein-DNA binding mode. In the first stage, a well-known machine leaning approach has been used for prediction from amino acid sequences which uses support vector machine with features created by the evolutionary profile of the proteins [43,44]. The evolutionary profile of position-specific scoring matrices (PSSM) is computed by PSI-BLAST [45] against the NR database for a protein sequence. In addition, in order to keep evolutionary information of neighborhood residues information, we use the principle of sliding window to calculate the backward (or/and forward) metrics over a limited region of the received sequence. For each residue in a protein sequence, we use a sliding window of size 11 to describe neighborhood information; therefore, we have a 11 * 21 = 231 dimension feature factor in addition to the 20 amino acids and a boundary flag. In the end, we used LIBSVM [39] as predictor to predict DNA-binding residues. The best parameters selected for DNA-binding residues prediction is decided by leave-one-out cross validation (LOOCV).

In second stage, protein-DNA binding mode is predicted by using the prediction results of the previous stage. In Table 6, DNA-binding domains recognized by Pfam [46] will be classified into five binding modes, including zipper-type, helix-turn-helix (HTH), zinc-coordinating, β-hairpin/ribbon, and others. As shown in Table 7, there are 28 features for protein-DNA binding mode prediction including the information of non-specific binding residues, predicted secondary structure elements, and the number of total residues. The secondary structure elements for each protein structure in the training data are determined by DSSP program [47]. Because this predictor is a sequence based predictor to identify protein-DNA binding mode, the secondary structure elements for each protein structure in testing data (query protein) are predicted by PSIPRED [48]. In the training dataset, we used only the residue information in DNA-binding domain detected by Pfam server.

**Table 6:** Protein-DNA binding modes and their corresponding Pfam domains

Protein-DNA Binding mode	Pfam Domain
Zipper-type	HLH
	bZIP_1
	bZIP_2

Helix-turn-helix	HTH_AraC
	TetR_N
	Trp_repressor
	Homeobox
	E2F_TDP
	GntR
	Fe_dep_repress
	Ets
	HTH_3
	Sigma70_r4
	LacI
	Fork_head
	Resolvase
	Pou

Zinc-coordinating	zf-C4
	zf-C2H2
	Zn_clus
	GATA
	P53

β-hairpin/ribbon	MetJ
	RHH_1
	Arc

Others	SRF-TF
	Runt
	PAX
	RHD
	Autoind_bind
	cNMP_binding

**Table 7:** Illustration of feature set for protein-DNA binding mode prediction

Feature title	Feature descriptions
Class label	5 protein-DNA binding modes
	1. zipper-type
	2. helix-turn-helix (HTH)
	3. zinc-coordinating
	4. β-hairpin/ribbon
	5. others

Non-specific binding	20 dimensions of amino acid
	3 dimensions of secondary structure elements
	# of binding residues

Protein chain information	3 dimensions of secondary structure elements
	# of total residues in a protein chain

### Predictor performance measures

The predictions made for the testing instances are compared with the defined class labels (binding or non-binding) to evaluate the predictor. The accuracy is defined as

where TP is the number of true positives (binding residues with positive predictions); TN is the number of true negatives (non-binding residues with negative predictions); FP is the number of false positives (non-binding residues but predicted as binding sites) and FN is the number of false negatives (binding residues but predicted as non-binding sites). Since the data for DNA-binding residue prediction is skewed, the accuracy alone may be misleading. The predictor can achieve 85% accuracy by simply predicting all residues as negative for datasets where the positive to negative sample ratio is 1:10. Therefore, we focus on the specificity and sensitivity of the predictions, which are defined as follows:

The sensitivity is used to measure the prediction capability of positive samples; the specificity is used to measure the prediction capability of negative samples. In addition, precision and F-measure are also defined as follows:

## List of abbreviations used

GO: Gene Ontology; LOOCV: Leave-one-out cross-validation; NDB: Nucleic Acid Database; PDB: Protein Data Bank; SVM: Support vector machine; TFs: Transcription factors;

## Competing interests

The authors declare that they have no competing interests.

## Authors' contributions

YFH and CCH developed and implemented the overall framework and drafted the manuscript; YCL provided valuable suggestion on dealing with dataset and protein-DNA complexes. YFH and CKH edited the manuscript and introduced the problem initially. YJO gave suggestions on fundamental knowledge of machine learning. All authors have read and approved the final manuscript.

## Note

Other papers from the meeting have been published as part of *BMC Bioinformatics* Volume 10 Supplement 15, 2009: Eighth International Conference on Bioinformatics (InCoB2009): Bioinformatics, available online at http://www.biomedcentral.com/1471-2105/10?issue=S15.
